# INTIMATE PSYCOLOGICAL VIOLENCE ASSOCIATED WITH ALCOHOL USE

**DOI:** 10.1192/j.eurpsy.2023.1908

**Published:** 2023-07-19

**Authors:** J. S. Castillo, M. M. Llamuca, V. Valdez, C. Santana

**Affiliations:** 1 Universidad Católica Santiago de Guayaquil, guayaquil, Ecuador; 2 Universidad Catolica de Santiago de Guayaquil, Universidad Católica Santiago de Guayaquil, guayaquil, Ecuador

## Abstract

**Introduction:**

Alcohol and Drug use is an important Public Health problem, it has a negative impact on the cognitive and individual behavior. IPV has been frequently connected to drug alcohol and drug use.

**Objectives:**

The aim of this study is to explore the relationship between IPV and alcohol abuse.

**Methods:**

An observational and descriptive study was carried out using the internet platform “google forms”, after requesting the informed consent of each of the participants, we collected the data of affiliation and the Test of Identification of Disorders due to Alcohol Consumption (AUDIT) with the Drug Use Disorders Identification Test (DUDIT) was applied to identify individuals with a pattern of harmful consumption of substances harmful to health as alcohol and drugs.

**Results:**

The total sample was 851, it was classified according to age, sex, marital status, level of education, urban or rural population, and whether they had suffered any type of violence associated with alcohol abuse. The mean age: 26 - 27 years old. The results according to the AUDIT TEST: 562 (66%) reported not having suffered any type of violence, 289 (34%) reported some type of violence, 157 (18%) psychological violence, 10 (1%) psychological violence and patrimonial violence, 16 (2%) psychological and sexual violence.

**Conclusions:**

This study established an important population who has suffered Psychological Violence. Other types of Violence has been determined. it is important to highlight that these results showed an important information to work on prevention. Further studies are an urgent need.

**Disclosure of Interest:**

None Declared

